# Supplementary material for: High-dimensional communication on etchless lithium niobate platform with photonic bound states in the continuum
Source: Nat Commun. 2020 May 25;11:2602. doi: 10.1038/s41467-020-15358-x (PMC7248070; doi:10.1038/s41467-020-15358-x)
Supplement: Supplementary file 2 — Supplementary Information [file 41467_2020_15358_MOESM2_ESM.pdf]

Supplementary Information for "High-dimensional communication on etchless lithium niobate platform with photonic bound states in the continuum"

Zejie Yu, Yeyu Tong, Hon Ki Tsang, and Xiankai Sun*

Department of Electronic Engineering, The Chinese University of Hong Kong, Shatin, New Territories, Hong Kong

**Corresponding author: xksun@cuhk.edu.hk*

Supplementary Note 1: BICs of different orders

Solving the Schrödinger equation for a quantum system with a finite potential well, one obtains the eigenstates with their energy eigenvalues lying inside the potential well. These eigenstates can be square integrable and are known as bound states of the quantum system. The other eigenstates with their energy eigenvalues outside the potential well are not square integrable. These eigenstates are known as continuous states, which are extended to infinity. Taking the analogy of the Schrödinger equation and the Helmholtz equation[1,2], the refractive index n of a material determines the potential for photons inside that material as $-n^2k_0^2$, where k_0 is the wave number of light in the vacuum. Therefore, a higher (lower) effective refractive index leads to a lower (higher) potential for photons.

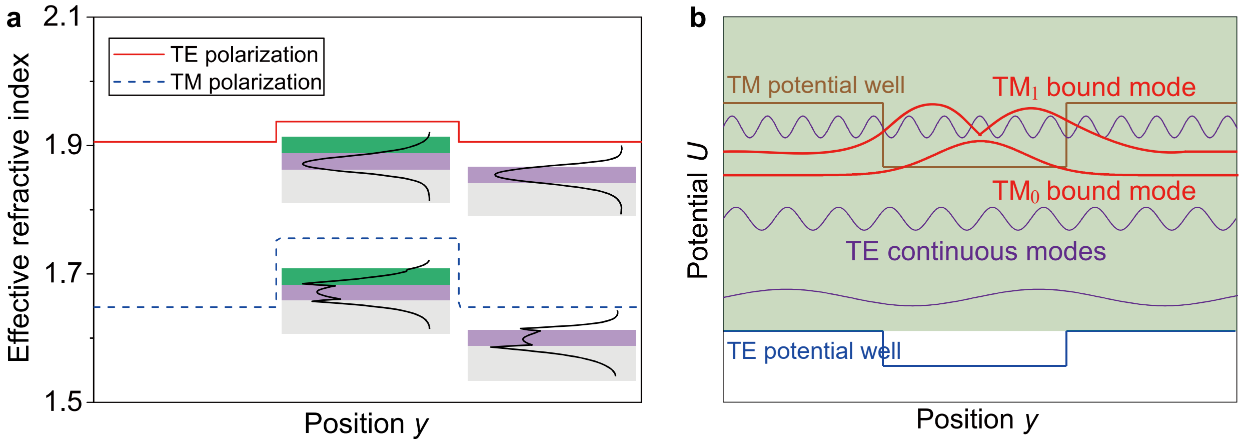

Supplementary Figure 1. (a) Effective refractive index distributions for the TE and TM polarizations in the waveguide in Fig. 1a. The insets show the |**E**| field distributions of the TE and TM modes of the $LiNbO_3$-on-insulator substrate with or without the low-refractive-index polymer atop. (b) Photonic potential distribution of the hybrid waveguide structure shown in Fig. 1a. The blue and brown lines represent the potential wells for the TE and TM polarizations, respectively. The TM bound modes (red lines) lie in the TE continuous spectrum.

Supplementary Fig. 1a plots the effective refractive index distributions for the transverse electric (TE) (red solid) and transverse magnetic (TM) (blue dashed) polarizations at the wavelength of 1.55 μm, where the effective refractive index for the TM polarization lies below that for the TE polarization. The insets of Supplementary Fig. 1a show the |**E**| field distributions of the TE and TM modes of the $LiNbO_3$-on-insulator substrate with or without the low-refractive-index polymer

atop. The thicknesses of the low-refractive-index polymer waveguide and $LiNbO_3$ layer are 500 and 400 nm, respectively.

Supplementary Fig. 1b plots the photonic potential distributions for the TE and TM polarizations in the hybrid waveguide structure in Fig. 1a, based on the effective refractive index distributions in Supplementary Fig. 1a. The potential well for the TM polarization lies above that for the TE polarization. Therefore, the TM bound modes localized in the TM potential well lie in the TE continuous spectrum. Under this condition, the inevitable coupling between the TM bound modes and the TE continuous modes causes optical loss to the TM bound modes. Defying this conventional wisdom, bound states in the continuum (BICs) refer to a type of eigenstates whose energy eigenvalues are above the potential well yet the corresponding wavefunctions are square integrable. Therefore, harnessing the BICs in the hybrid waveguide structure in Fig. 1a will enable lossless light guidance and propagation of the TM bound modes in the TE continuum.

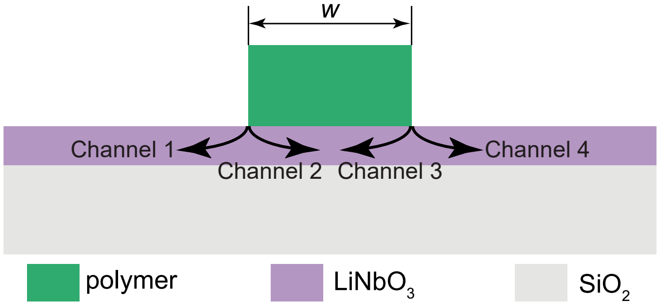

Supplementary Figure 2. Radiation channels of the TM bound mode to the TE continuum.

In a low-refractive-index waveguide on a high-refractive-index $LiNbO_3$-on-insulator substrate, the interaction between the TM bound modes (Figs. 1b and 1c) and the TE continuous modes (Fig. 1d) in the $LiNbO_3$ layer is generally inevitable, which causes optical loss to the TM bound modes. The loss of the TM bound mode to the TE continuum occurs at the two waveguide edges as illustrated in Supplementary Fig. 2. The loss at each edge originates from the coupling of the TM bound mode with the left-going (Channels 1 and 3) and right-going (Channels 2 and 4) TE continuous modes. If the losses via Channels 1 (2) and 3 (4) interfere destructively and cancel each other out, then the total loss of the TM bound mode to the TE continuum can be reduced to zero, leading to a lossless TM bound mode which is the desired BIC. The interference of losses via Channels 1 (2) and 3 (4) depends on the phase difference caused by the finite width of the waveguide, so the BIC can be obtained just by optimizing the waveguide width w. It should be noted that the above analysis applies generally to the TM bound modes of any order. The only difference among the TM BIC modes of different orders lies in their coupling with the TE continuous modes, so they require different waveguide widths to achieve the zero propagation loss. The propagation length of the TM bound modes in a straight waveguide can be expressed as[3]

$$L \propto \frac{w^2}{\sin^2(k_y w/2)}, \quad (1)$$

with k_y the y component of the wave number of the TE continuous mode which matches that of the TM bound mode. Therefore, the propagation length L corresponding to the BICs approaches infinity when $k_y w$ is equal to a multiple of 2π.

We calculated the propagation loss for the straight waveguide shown in Fig. 1a with a finite-element method in COMSOL[4]. We set the refractive indices of $LiNbO_3$ $n_o = 2.21$ and $n_e = 2.13$, and the refractive index of polymer 1.54. For a 500-nm-thick low-refractive-index polymer waveguide on a 400-nm $LiNbO_3$-on-insulator substrate, the simulated propagation loss for light as a function of both the waveguide width w and wavelength λ is shown in Supplementary Fig. 3. The propagation loss can reach zero within a large bandwidth for a certain combination of structural parameters.

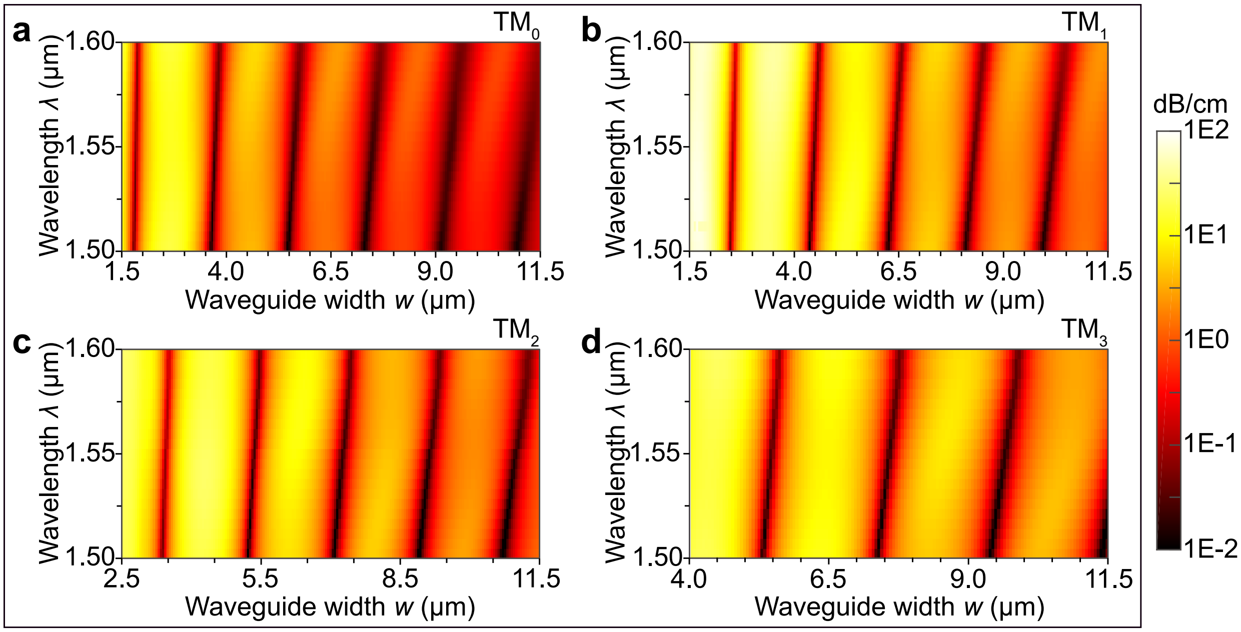

Supplementary Figure 3. Propagation loss of the TM_0 (a), TM_1 (b), TM_2 (c), and TM_3 (d) modes as a function of both the waveguide width w and wavelength λ.

Supplementary Note 2: Crosstalk between different mode channels

The influence of crosstalk on the eye diagrams is analyzed to show our fabricated mode (de)multiplexer can work in real applications. In our experiment, the crosstalk for each channel was introduced by the other three channels. For example, the modulated signal in the TM_2–TM_2 channel could be influenced by the signals input into the TM_0, TM_1, and TM_3 channels, because the signals from the TM_0, TM_1, and TM_3 channels could also be coupled slightly into the TM_2 channel. These undesired signals would affect the purity of signal in the TM_2–TM_2 channel. The influence of such crosstalk on eye diagrams was numerically calculated based on the experimentally measured eye diagrams in Fig. 7b and the crosstalk in Figs. 5b–5e. Supplementary Figs. 4a–4e plot the simulated eye diagrams for the TM_0, TM_1, TM_2, and TM_3 output channels with no crosstalk, with crosstalk as the measured results in Figs. 5b–5e, with crosstalk of −10, −7, and −5 dB between any two channels, respectively. A comparison between Supplementary Figs. 4a and 4b concludes that the experimental crosstalk has negligible influence on the measured eye diagrams. Supplementary Figs. 4c–4e show that the eye diagrams start to be affected when the crosstalk is −7 dB between any two channels and have almost closed eyes when the crosstalk

increases to −5 dB between any two channels. These results indicate that our fabricated mode (de)multiplexer can support 40-Gbps/channel data transmission in real applications.

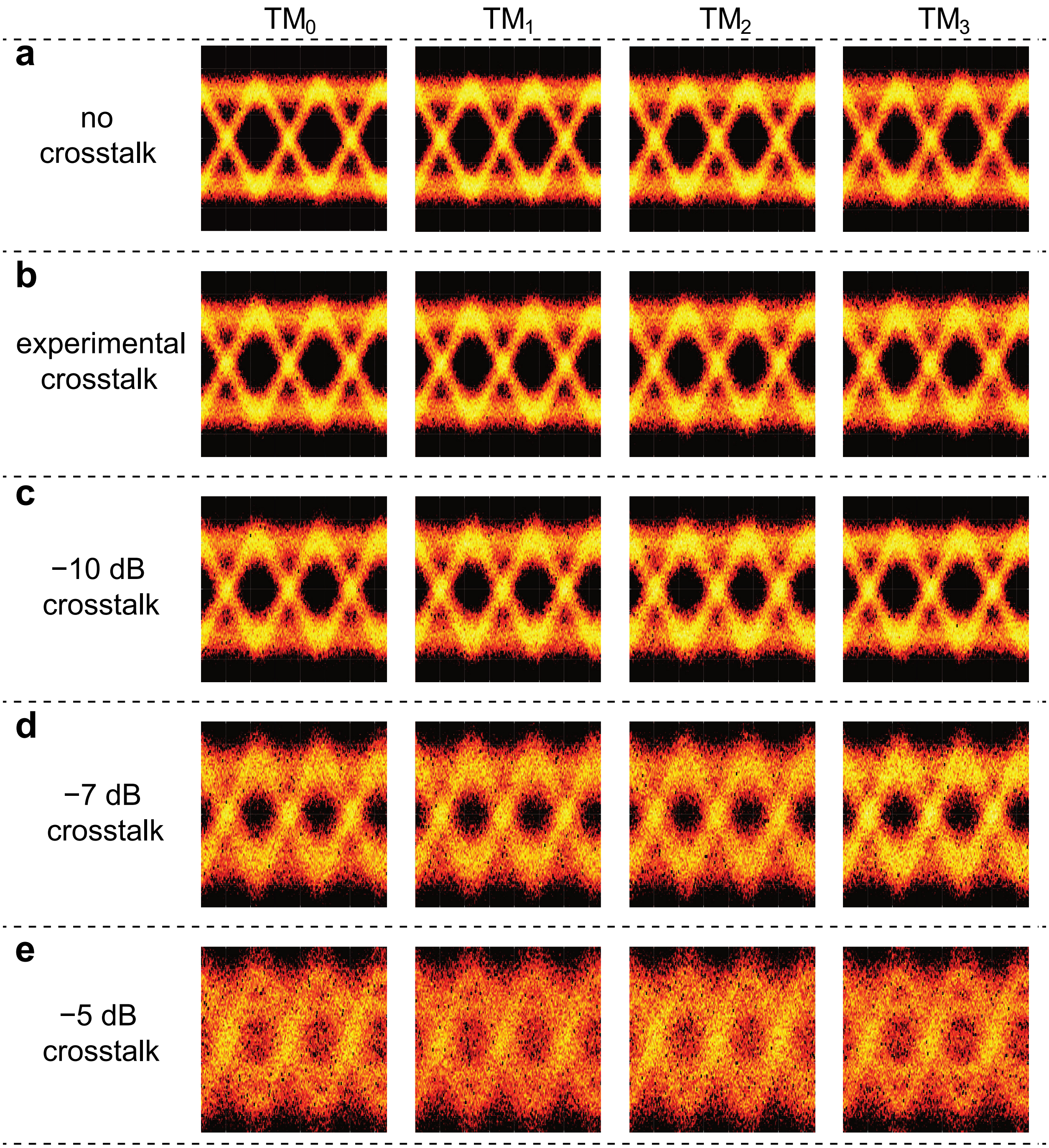

Supplementary Figure 4. (a)–(e) Simulated eye diagrams for the TM_0, TM_1, TM_2, and TM_3 output channels with no crosstalk (a), with crosstalk as the measured results in Figs. 5b–5e (b), with crosstalk of −10 dB (c), −7 dB (d), and −5 dB (e) between any two channels.

Supplementary Note 3: Details of fabricated devices and measurement

Supplementary Fig. 5a is an optical microscope image of a fabricated device with two grating couplers connected by a straight waveguide. Supplementary Fig. 5b is a scanning electron microscope image zoomed in at the grating coupler, which has a grating period of 1.15 μm and duty cycle of 0.5. Supplementary Fig. 5c plots the measured optical transmission spectrum of a single grating coupler, which shows that the minimal insertion loss of a single grating coupler is less than 12 dB.

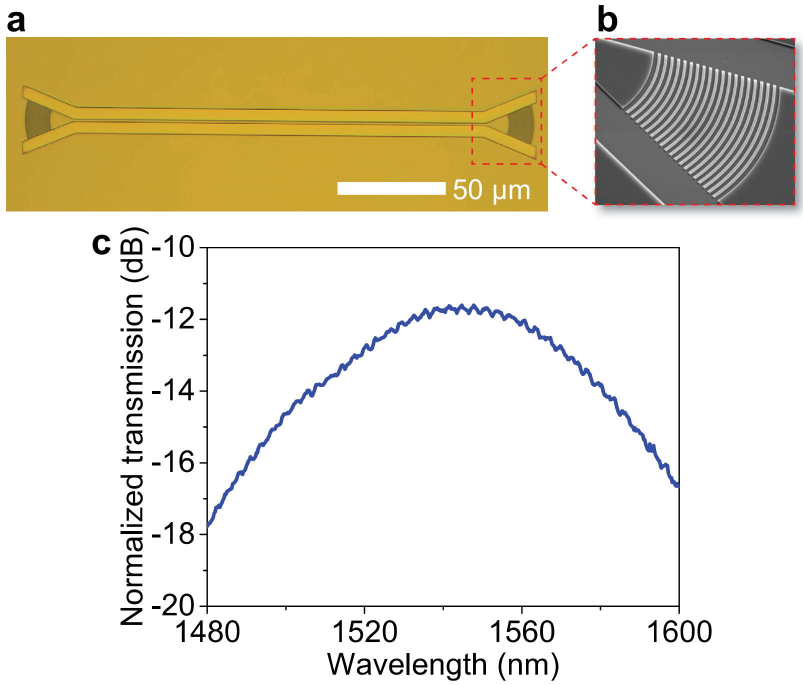

Supplementary Figure 5. (a) Optical microscope image of fabricated grating couplers connected by a straight waveguide. (b) Scanning electron microscope image of a grating coupler. (c) Measured spectrum of optical transmission through a single grating coupler.

The structural parameters for the fabricated TM_0–TM_i (i = 1, 2, and 3) multimode directional couplers (Fig. 4a) are labeled in Supplementary Fig. 6a. The width w_0, bend radius R, and bend angle θ of the waveguides supporting the TM_0 mode are 1.82 μm, 150 μm, and 30°, respectively. The gap g_i between the two waveguides and the width w_i of the main waveguide are (g_i, w_i) = (0.55 μm, 4.42 μm), (0.55 μm, 7.02 μm), and (0.45 μm, 9.65 μm) for the TM_0–TM_1, TM_0–TM_2, and TM_0–TM_3 multimode directional couplers, respectively. The coupling lengths L_i were determined experimentally to maximize the coupling efficiency for the high-order modes at the wavelength of 1.55 μm, which were found to be L_1 = 140 μm, L_2 = 175 μm, and L_3 = 175 μm for the TM_0–TM_1, TM_0–TM_2, and TM_0–TM_3 multimode directional couplers, respectively. Since the 4-channel mode (de)multiplexer (Fig. 5a) was constructed from the experimentally optimized TM_0–TM_1, TM_0–TM_2, and TM_0–TM_3 multimode directional couplers, its structural parameters in the mode-coupling sections follow those of the individual multimode directional couplers. In the sections between the mode-coupling sections, the width of the main waveguide is varied gradually to ensure adiabatic transformation of all the supported modes.

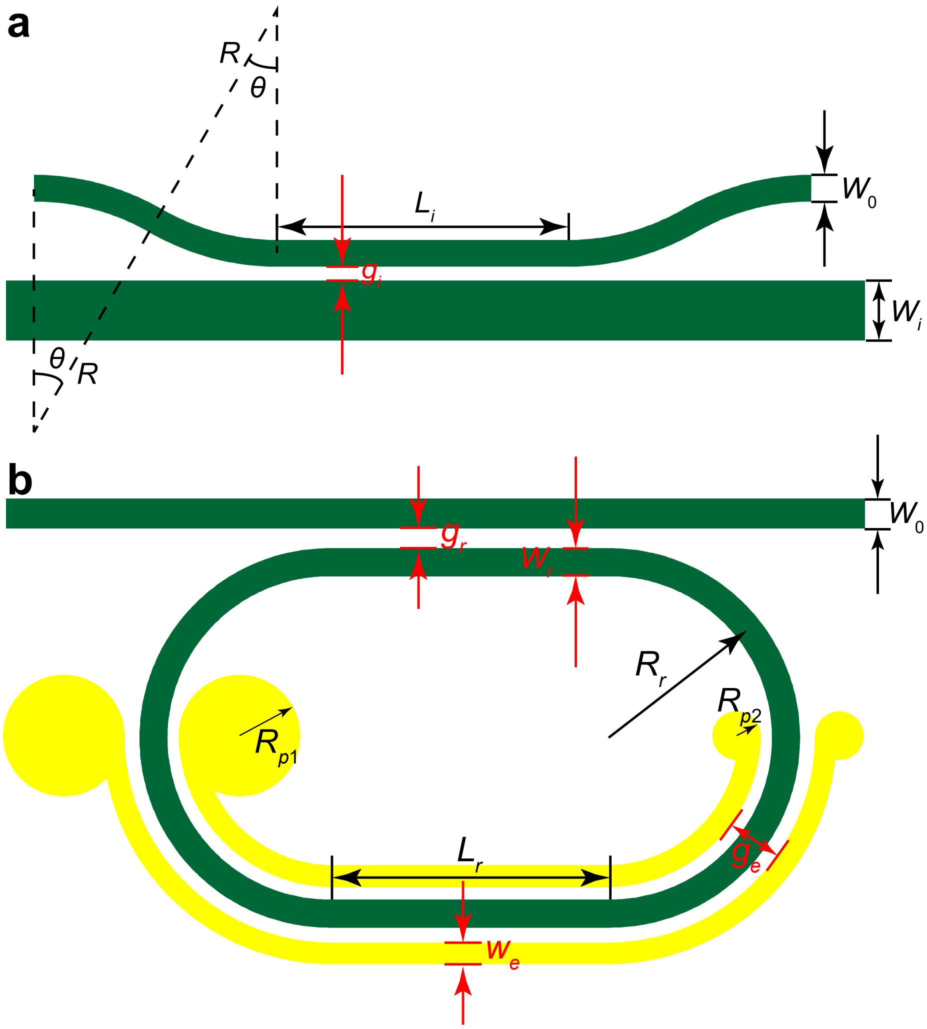

Supplementary Figure 6. (a) Illustration of a multimode directional coupler with the labeled structural parameters. (b) Illustration of an electro-optic modulator with the labeled structural parameters.

For the fabricated mode (de)multiplexer integrated with electro-optic modulators shown in Fig. 6a, the structural parameters for the mode (de)multiplexer are identical to those of the device in Fig. 5a. The structural parameters for the electro-optic modulators are labeled in Supplementary Fig. 6b. An electro-optic modulator consists of a bus waveguide and a racetrack microcavity with a pair of electrodes. The gap g_r between the bus waveguide and the straight section of the racetrack microcavity is 0.8 μm. The waveguide width w_r, the length L_r of the straight section, and the radius R_r of the bent section of the racetrack microcavity are 1.82 μm, 100 μm, and 150 μm, respectively. The width w_e of the electrode stripes is 20 μm. The gap g_e between the two electrode stripes is 10 μm. The radius R_{p1} of the left pair of electrode pads (for landing the microwave probe) is 50 μm, and the radius R_{p2} of the right pair of electrode pads (for avoiding patterns of sharp corners and minimizing the charging effect during electron-beam lithography in device fabrication) is 20 μm. It should be noted that the electrodes were not optimized for high modulation speed.

For the measurement of on-chip electro-optic modulation and mode (de)multiplexing in Fig. 6b, light at the wavelength of ~1.55 μm from a tunable semiconductor laser (Yenista Tunics-T100S-

HP) was amplified by an EDFA (Amonics AEDFA PA-35-B-FA) and then sent through a fiber polarization controller before being coupled into the device under test. Meanwhile, an electrical driving signal with a peak voltage of 30 V from a signal generator (R&S SMA100B) was applied to the device under test via a microwave probe. The light transmitted through the device was collected by a high-speed photodetector (Newport 1811FC) which converted the detected optical signal into the electrical domain for monitoring on an oscilloscope (R&S RTC1002). For the measurement of high-dimensional data transmission through the fabricated 4-channel mode (de)multiplexer in Fig. 7a, light at the wavelength of ~1.55 μm from a tunable semiconductor laser (Yenista Tunics-T100S-HP) was amplified by an EDFA (Amonics AEDFA PA-35-B-FA) and then sent through a fiber polarization controller, followed by an electro-optic amplitude modulator (Fujitsu FTM7937EZ) which was driven by a pseudorandom binary sequence generator (Centellax TG1P4A) at a bit rate of 40 Gbps. The modulated light was sent through a second fiber polarization controller before being coupled into the device under test. The light transmitted through the device was amplified by a second EDFA (Amonics AEDFA PA-35-B-FA) and then collected by a high-speed photodetector (Finisar MPRV1331A) which converted the detected optical signal into the electrical domain for monitoring on a high-speed oscilloscope (Tektronix DSA8300).

Supplementary References

1. Longhi, S. Quantum-optical analogies using photonic structures. *Laser Photon. Rev.* **3**, 243–261 (2009).
2. Dragoman, D. & Dragoman, M. *Quantum-Classical Analogies*. (Springer Science & Business Media, 2013).
3. Zou, C.-L. *et al.* Guiding light through optical bound states in the continuum for ultrahigh-*Q* microresonators. *Laser Photon. Rev.* **9**, 114–119 (2015).
4. COMSOL Multiphysics® v. 4.3a. www.comsol.com.
